# Portal hypertension in patients with hepatocellular carcinoma and immunotherapy: prognostic relevance of CT-morphologic estimates

**DOI:** 10.1186/s40644-023-00558-7

**Published:** 2023-04-25

**Authors:** Lukas Müller, Simon J. Gairing, Friedrich Foerster, Arndt Weinmann, Jens Mittler, Fabian Stoehr, Dirk Graafen, Christoph Düber, Peter R. Galle, Roman Kloeckner, Felix Hahn

**Affiliations:** 1 Department of Diagnostic and Interventional Radiology, University Medical Center of the Johannes Gutenberg University Mainz, Langenbeckstr. 1, Mainz, 55131 Germany; 2 Department of Internal Medicine I, University Medical Center of the Johannes Gutenberg University Mainz, Mainz, Germany; 3 Department of General, Visceral and Transplant Surgery, University Medical Center of the Johannes Gutenberg University Mainz, Mainz, Germany; 4 Institute of Interventional Radiology, University Hospital Schleswig-Holstein-Campus Luebeck, Luebeck, Germany

**Keywords:** Hepatocellular carcinoma, Prognosis, Immunotherapy, Portal hypertension

## Abstract

**Background:**

Clinically significant portal hypertension (CSPH) has been identified as an important prognostic factor in patients with hepatocellular carcinoma (HCC) undergoing curative treatment. This study aimed to assess PH estimates as prognostic factors in patients with HCC treated with immunotherapy.

**Methods:**

All patients with HCC treated with an immunotherapeutic agent in first or subsequent lines at our tertiary care center between 2016 and 2021 were included (*n* = 50). CSPH was diagnosed using the established PH score for non-invasive PH estimation in pre-treatment CT data (cut-off ≥ 4). Influence of PH on overall survival (OS) and progression-free survival (PFS) was assessed in uni- and multivariable analyses.

**Results:**

Based on the PH score, 26 patients (52.0%) were considered to have CSPH. After treatment initiation, patients with CSPH had a significantly impaired median OS (4.1 vs 33.3 months, *p* < 0.001) and a significantly impaired median PFS (2.7 vs 5.3 months, *p* = 0.02). In multivariable Cox regression, CSPH remained significantly associated with survival (HR 2.9, *p* = 0.015) when adjusted for established risk factors.

**Conclusions:**

Non-invasive assessment of CSPH using routine CT data yielded an independent prognostic factor in patients with HCC and immunotherapy. Therefore, it might function as an additional imaging biomarker to detect high-risk patients with poor survival and possibly for treatment decision making.

## Introduction

Hepatocellular carcinoma (HCC) is the most common primary liver cancer and a major cause for cancer-related deaths worldwide [1, 2]. Systemic therapy is the mainstay treatment for patients in advanced tumor stages and for patients that experienced a failure of previous surgical or locoregional treatment [3]. For these patients, treatment with immunotherapeutic agents has gained importance over the recent years: the results of the IMbrave150 phase III trial established immunotherapy with atezolizumab plus bevacizumab (a + b) as new first-line therapy for systemic therapy-naïve patients with advanced-stage HCC. In the study, a + b showed a median overall survival (OS) of 19.2 months compared to 13.4 months for sorafenib [4], which was recently confirmed in a large real-world data set [5]. Currently, several ongoing trials are investigating the potential of several other immunotherapeutic agents, both for the treatment of advanced HCC and for the potential treatment of patients with earlier-stage tumors [6–8].

However, the IMbrave150 trial excluded patients with a history of varices-related bleeding in the last 6 months or untreated bleeding-prone varices [9]. A systematic review including phase II studies which evaluated bevacizumab monotherapy in HCC reported a gastrointestinal bleeding rate of 10% (predominantly due to esophageal varices, [10]). Thus, it is of pivotal importance to thoroughly assess the risk of portal hypertension-related bleeding in patients undergoing immunotherapy [11]. In addition, clinically significant portal hypertension (CSPH) itself leads to a higher risk of hepatic decompensation and negatively influences the overall survival (OS) of patients with HCC undergoing resection or locoregional treatment [12–14]. The current gold standard to detect CSPH is the direct measurement of the hepatic venous pressure gradient (HVPG, [15]). However, HVPG measurement is not available for most patients in clinical routine due to its invasive nature. Therefore, non-invasive PH assessment using routine diagnostic imaging may be a useful and readily available tool to risk stratify these patients. Recently, Kihira et al. developed an imaging-derived PH score [16]. A cut-off ≥ 4 yielded the best discriminator for detecting patients with an HVPG ≥ 10 mmHg indicating CSPH. Although non-invasive PH estimation can be performed using routinely acquired cross-sectional imaging, literature on the prognostic relevance of CSPH in patients with HCC and systemic treatment remains unclear. Particularly, the prognostic relevance of CT-morphologic estimates of PH for patients with HCC treated with immunotherapy has not been evaluated so far.

This study aimed to evaluate the association of CT-based PH estimation with survival in patients with HCC undergoing immunotherapy and thus to investigate the potential use of estimates for PH as novel imaging biomarkers in these patients.

## Methods

The responsible Ethics Committee (Medical Association of Rhineland Palatinate, Mainz, Germany) approved this study (permit number 837.199.10). Informed consent was waived due to the retrospective nature of the study.

### Patients

All patients who presented themselves in our HCC outpatient clinic between May 2016 and October 2021 for the initiation of immunotherapy were considered for inclusion. The inclusion criteria were: (1) age above 18 years, (2) HCC diagnosis based on the histopathology or image-derived EASL criteria, (3) immunotherapy in first or further lines, (4) available CT imaging prior to immunotherapy, (5) adequate image-quality, and (6) available demographical, clinical, and laboratory data at initiation of the immunotherapy and during follow-up. Of the scanned 64 patients, a total of 50 (78.1%) patients fulfilled all inclusion criteria.

### Diagnosis, treatment and follow-up

Histological or image-derived EASL criteria were used for the diagnosis of HCC [3, 17]. Initiation of immunotherapy was based on an interdisciplinary tumor board decision, which consisted of hepatologists/oncologists, diagnostic and interventional radiologists, visceral surgeons, pathologists, and radiation therapists. Prior to the initiation of immunotherapy and during follow-up, all patients underwent a triple-phase contrast-enhanced computed tomography (CT) scan with a late arterial, a portal venous, and a delayed phase. All CT images were obtained using a 256-slice CT scanner (iCT, Philips, Eindhoven, the Netherlands – collimation 128 × 0.625 mm) or a clinical photon-counting detector CT scanner (Naeotom Alpha®, Siemens Healthineers, Erlangen, Germany – collimation 144 × 0.4 mm). For the 256-slice CT scanner, tube voltages were 80, 120, and 120 kVp for late arterial, portal venous, and delayed phase acquisition, respectively. For the photon-counting detector CT and all contrast phases, scans were performed in QuantumPlus mode at 120 kVp obtaining full spectral information. Tube current was modulated dependent on the body characteristics of the patient using the standard algorithm of the vendors. Standard vendor specific solutions and recommendations were used for kernel selection and iterative reconstruction. Iopromide was used as iodinated contrast medium (Ultravist® 370, Bayer Vital, Leverkusen, Germany). Injection protocol consisted of a single-bolus contrast medium injection (volume: 120 ml, flow: 5 ml/s and iodine flux: 1.9 gI/s) followed by a saline bolus (volume: 50 ml, flow: 4 ml/s). Late arterial phase imaging was timed using bolus tracking in the proximal abdominal aorta with a threshold of 100 HU signal increase and 13 s post-threshold delay. Portal venous phase and delayed phase scans were acquired after a delay of 50 s and 180 s, respectively. During post-processing, images with a slice thickness of 1 mm and 3 mm were reconstructed in the axial orientation and in sagittal and coronal views. Follow-up consisted of clinical examination, blood sampling, and cross-sectional imaging, which was typically repeated every 6 to 12 weeks. Follow-up was performed until November 2022 (one year after the last patient was included).

### CT-morphologic estimates of portal hypertension

We used the previously reported criteria of the PH score, which was originally developed for patients with cirrhosis [16]. This simple score has shown a high correlation with invasive HVPG measurements and is additionally easy to apply in clinical routine. As part of the score the following parameters were assessed: presence of ascites, craniocaudal spleen size, and presence of varices. These parameters were further categorized as reported in the original study of the PH score (Table 1) [16]. The score was assessed by two radiologists with three and five years of experience in liver imaging. In case of disagreement, consensus reading was performed by an experienced third reader with more than eight years of experience in liver imaging. The readers had information on the clinical history of the patient but were blinded for the clinical outcome of the patients. All parameters of the PH score were evaluated in venous phase scans.

**Table 1:** Classification and score distribution for ascites, spleen size, and varices according to the PH score [16]

Variable	Valuation	Description
Ascites	0 points	No ascites
	1 point	Minimal perihepatic or perisplenic fluid
	2 points	Intraperitoneal fluid with no significant abdominal wall distension
	3 points	Fluid causing significant abdominal wall distension
Spleen size	0 points	Size less than 130 mm
	1 point	Size between 130 and 150 mm
	2 points	Size between 151 and 200 mm
	3 points	Size more than 200 mm
Varices	0 points	Absence of varices
	1 point	Varices in one location^a^
	2 points	Varices in two locations^a^
	3 points	Varices in more than two locations^a^

^a^Five locations are screened for the presence of varices or collaterals: gastric, paraesophageal, splenorenal, paraumbilical, and other(s)

### Statistical analysis

Statistical analyses and graphic design were performed in R 4.0.3 (A Language and Environment for Statistical Computing, R Foundation for Statistical Computing, http://www.R-project.org; last accessed 30 11 2022). Continuous data were reported as median and interquartile range. Categorical and binary baseline parameters were reported as absolute numbers and percentages. Categorical variables were compared using the chi-square test. Otherwise, Wilcoxon-Mann–Whitney test was performed. Survival analyses and creation of the Kaplan–Meier curves were performed with the packages "survminer" and "survival" (https://cran.r-project.org/package=survminer, https://CRAN.R-project.org/package=survival, accessed 30 11 2022). For all patients, overall survival (OS) and progression-free survival (PFS) were calculated starting from the initiation of the treatment. Log-rank testing was used for the comparison of survival times. Cox proportional hazards regression models assessing hazard ratios (HRs) and corresponding 95% confidence intervals (CIs) were used to determine the effect of the risk stratification. *P*-values less than 0.05 were considered significant.

## Results

### Patients’ characteristics

Among the 50 included patients, 40 (80.0%) were men and the median age was 68 (62 – 73) years. Table 2 provides a detailed overview of the patients’ baseline characteristics.

**Table 2:** Baseline characteristics of the patient cohort

Parameter	All patients (*n* = 50)	PH score ≤ 3 (*n* = 24)	PH score ≥ 4 (*n* = 26)	*P* value
Age, Median (IQR)	68 (62 – 73)	70 (65 – 76)	64 (61 – 71)	0.025
Sex, n (%)				0.832
Female	10 (20.0)	4 (16.7)	6 (23.1)	
Male	40 (80.0)	20 (83.3)	20 (76.9)	
Etiology of cirrhosis, n (%)				0.816
Alcohol	19 (38.0)	9 (37.5)	10 (38.5)	
Viral	7 (14.0)	2 (8.3)	5 (19.2)	
Other	11 (22.0)	6 (25.0)	5 (19.2)	
No cirrhosis	13 (26.0)	7 (29.2)	6 (23.1)	
Child–Pugh stage, n (%)				0.266
A	25 (50.0)	14 (58.3)	11 (42.3)	
B	10 (20.0)	3 (12.5)	7 (26.9)	
C	2 (4.0)	0	2 (7.7)	
No cirrhosis	13 (26.0)	7 (29.2)	6 (23.1)	
ECOG, n (%)				1.000
≤ 1	47 (94.0)	23 (95.8)	24 (92.3)	
2	3 (6.0)	1 (4.2)	2 (7.7)	
BCLC stage, n (%)				0.797
B	5 (10.0)	2 (8.3)	3 (11.5)	
C	42 (84.0)	21 (87.5)	21 (80.8)	
D	3 (6.0)	1 (4.2)	2 (7.7)	
Portal vein invasion, n (%)				0.991
Yes	26 (52.0)	13 (54.2)	13 (50.0)	
No	24 (48.0)	11 (45.8)	13 (50.0)	
Distant metastasis, n (%)				1.000
Yes	25 (50.0)	12 (50.0)	13 (50.0)	
No	25 (50.0)	12 (50.0)	13 (50.0)	
Focality of the liver lesions, n (%)				1.000
Unifocal	11 (22.0)	6 (25.0)	5 (19.2)	
Multifocal	39 (78.0)	18 (75.0)	21 (80.8)	
Sum of the size of the target lesions, mm, Median (IQR)	83 (51 – 135)	58 (45 – 122)	97 (59 – 149)	0.205
AFP, ng/ml, Median (IQR)	277 (16 – 4485)	84 (12 – 1467)	60 (1240 – 18,359)	0.071
Albumin, g/l, Median (IQR)	30 (27 – 33)	33 (30 – 36)	28 (25 – 30)	< 0.001
Bilirubin, mg/dl, Median (IQR)	1.5 (0.7 – 2.3)	0.8 (0.6 – 1.7)	2.2 (1.5 – 3.1)	< 0.001
INR, Median (IQR)	1.2 (1.1 – 1.3)	1.2 (1.1 – 1.4)	1.2 (1.1 – 1.3)	0.984
Creatinine, mg/dl	0.9 (0.7 – 1.1)	0.8 (0.7 – 1.0)	0.9 (0.7 – 1.2)	0.431
Immunotherapeutic agent, n (%)				
atezolizumab + bevacizumab	29 (58.0)	17 (70.8)	12 (46.2)	0.201
pembrolizumab	11 (22.0)	3 (12.5)	7 (26.9)	
nivolumab	10 (20.0)	4 (16.7)	7 (26.9)	
Line of systemic treatment, n (%)				0.107
First	29 (58.0)	17 (70.8)	12 (46.2)	
Second	11 (22.0)	5 (20.8)	6 (23.1)	
Third	10 (20.0)	2 (8.3)	8 (30.7)	
Previous therapy, n (%)				0.200
Yes	42 (84.0)	18 (75.0)	24 (92.3)	
No	8 (16.0)	6 (25.0)	2 (7.7)	

*ECOG* Eastern Cooperative Oncology Group, *BCLC* Barcelona Clinic Liver Cancer, *AFP* alpha-fetoprotein, *INR* International Normalized Ratio

### Distribution of the patients

A total of 26 (52.0%) of the patients had a PH score of ≥ 4 and were therefore considered to have CSPH. Among all patients, 30 (60.0%) had ascites, 21 (42.0%) had splenomegaly, and 40 (80.0%) had varices. Significant differences in the baseline characteristics for patients within the different PH score groups were observed for age, albumin, and bilirubin.

### Association of the PH score with OS

**Fig. 1:**
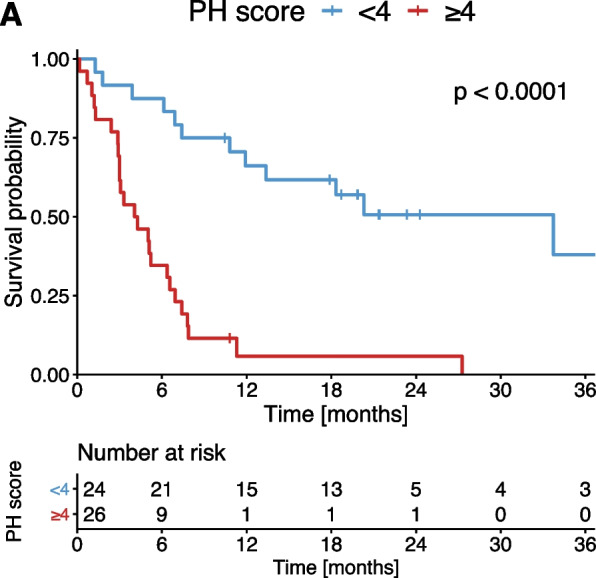
Kaplan–Meier estimates of OS stratified according to the PH score

Patients with a PH score of ≥ 4 had a median OS of 4.1 months and therefore significantly impaired survival compared to patients with a PH score < 4 (33.3 months, *p* < 0.001, Fig. 1). In multivariable Cox regression, CSPH remained significantly associated with survival (HR 2.9, *p* = 0.015) when adjusted for established risk factors (Table 3).

**Table 3:** Univariable and multivariable Cox regression analysis for OS

Analysis		Univariate			Multivariate		
**Score factor**		**HR**	**95% CI**	***p* value**	**HR**	**95% CI**	***p* value**
Age, years	> 65	0.7	0.4 – 1.3	0.290			
ECOG	> 1	1.6	0.5 – 5.1	0.460			
BCLC	C/D	0.8	0.3 – 2.0	0.570			
Child–Pugh	B/C	1.7	0.8 – 3.5	0.170			
AFP, ng/ml	> 200	2.0	1.1 – 3.9	**0.034**	1.9	1.0 – 3.8	0.069
Sum of the TL, mm	< 100	1.7	0.9 – 3.3	0.140			
Albumin, g/l	Cont.,	0.8	0.7 – 0.9	** < 0.001**	0.9	0.8 – 1.0	**0.030**
Bilirubin, mg/dl	Cont	1.9	1.5 – 2.5	** < 0.001**	1.4	1.0 – 1.9	0.080
INR	Cont	0.6	0.2 – 1.9	0.400			
Creatinine, mg/dl	Cont	1.0	0.4 – 2.5	0.970			
PH score	≥ 4	5.8	2.7 – 12.0	** < 0.001**	2.9	1.2 – 7.0	**0.015**

Outcome events: *n* = 38

*ECOG* Eastern Cooperative Oncology Group, *BCLC* Barcelona Clinic Liver Cancer Classification, *AFP* alpha-fetoprotein, *TL* target lesions, *INR* International Normalized Ratio

**Fig. 2:**
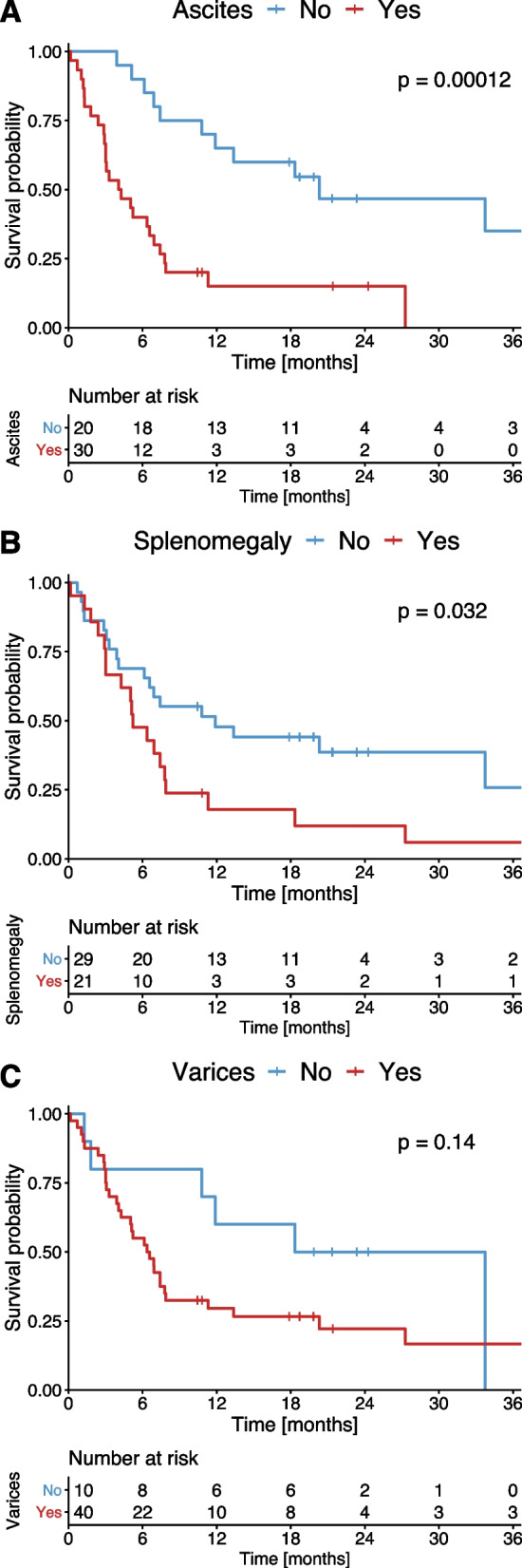
Kaplan–Meier estimates of OS stratified according to the presence of ascites (**A**), spleen size (**B**) and varices (**C**)

Of the individual score parameters, patients with ascites had a significantly impaired OS compared to patients without ascites (4.1 months vs 20.9 months, *p* < 0.001, Fig. 2A). Furthermore, patients with splenomegaly had an impaired OS compared to patients without splenomegaly (5.1 months vs 11.7 months, *p* = 0.032, Fig. 2B). The OS of patients with CT-detected varices did not differ significantly relative to patients without varices (6.4 months vs 25.7 months, *p* = 0.140, Fig. 2C).

Consequently, ascites and splenomegaly were the two significant factors of the PH score in univariate Cox regression analysis. In multivariate analysis comparing the two factors, presence of ascites remained the stronger prognostic factor (HR 3.8, *p* = 0.002, Table 4).

**Table 4:** Univariable and multivariable comparison for ascites, spleen size, and presence of varices

Analysis		Univariate			Multivariate		
**Score factor**		**HR**	**95% CI**	***p* value**	**HR**	**95% CI**	***p* value**
Ascites	Yes	4.0	1.9 – 8.5	** < 0.001**	3.8	1.6 – 8.6	**0.002**
Splenomegaly	Yes	2.0	1.0 – 3.9	**0.035**	1.1	0.5 – 2.3	0.750
Varices	Yes	1.9	0.8 – 4.7	0.145			

### Association of the PH score with PFS

**Fig. 3:**
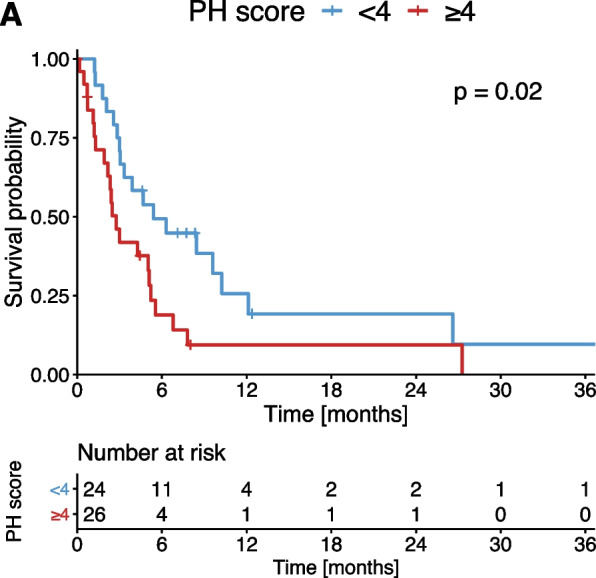
Kaplan–Meier estimates of PFS stratified according to the PH score

Patients with a PH score of ≥ 4 had a median PFS of 2.7 months which was significantly impaired compared to patients with a PH score < 4 (5.3 months, *p* = 0.020, Fig. 3).

While in univariate Cox regression analysis a high PH score was a prognostic factor for PFS, in multivariable analysis, a high PH score did not reach significance (HR 1.6, *p* = 0.312, Table 5).

**Table 5:** Univariable and multivariable Cox regression analysis for PFS

Analysis		Univariate			Multivariate		
**Score factor**		**HR**	**95% CI**	***p* value**	**HR**	**95% CI**	***p* value**
Age, years	> 65	0.9	0.5 – 1.7	0.830			
ECOG	> 1	0.9	0.3 – 3.1	0.920			
BCLC	C/D	1.9	0.6 – 6.2	0.310			
Child–Pugh	B/C	1.2	0.6 – 2.4	0.650			
AFP, ng/ml	> 200	1.7	0.9 – 3.3	0.089			
Sum of the TL, mm	< 100	2.0	1.0 – 3.8	**0.045**	2.0	1.0 – 4.3	**0.045**
Albumin, g/l	Cont.,	0.9	0.9 – 1.0	**0.015**	1.0	0.9 – 1.1	0.809
Bilirubin, mg/dl	Cont	1.5	1.2 – 1.9	** < 0.001**	1.5	1.0 – 1.9	**0.048**
INR	Cont	0.3	0.1 – 1.3	0.110			
Creatinine, mg/dl	Cont	1.3	0.5 – 3.2	0.580			
PH score	≥ 4	2.1	1.1 – 4.0	**0.023**	1.6	0.6 – 4.0	0.312

Outcome events: *n* = 40

*ECOG* Eastern Cooperative Oncology Group, *BCLC* Barcelona Clinic Liver Cancer, *AFP* alpha-fetoprotein, *INR* International Normalized Ratio

**Fig. 4:**
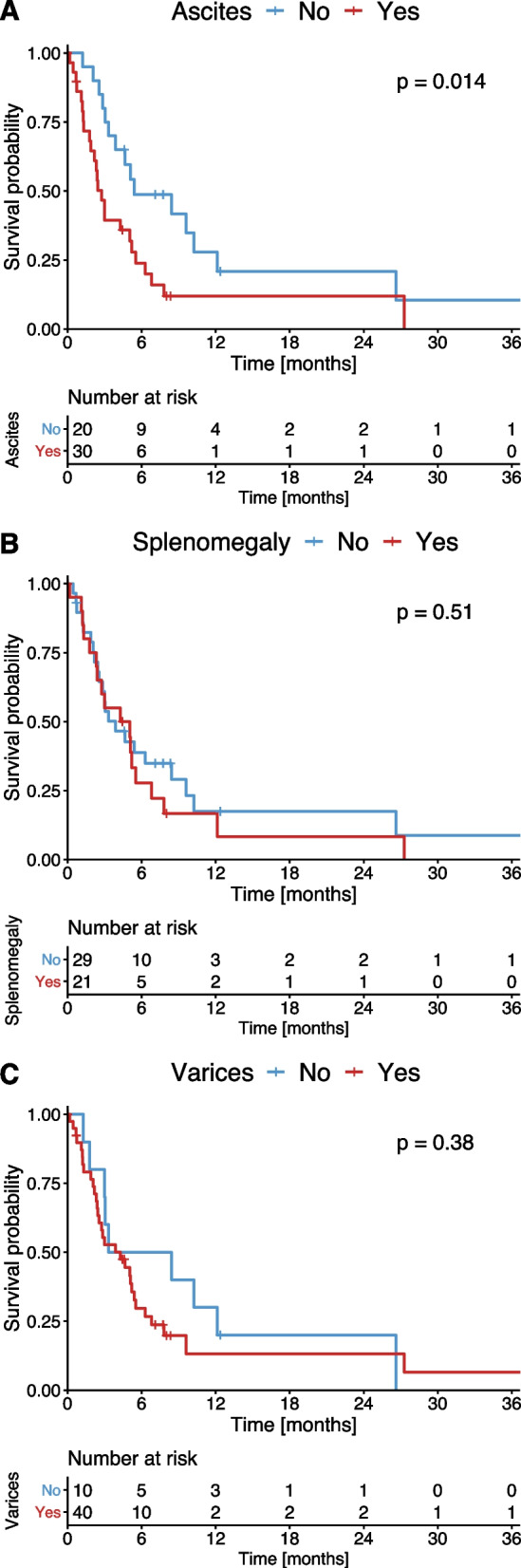
Kaplan–Meier estimates of PFS stratified according to the presence of ascites (**A**), spleen size (**B**) and varices (**C**)

Of the individual score parameters, patients showing presence of ascites had a significantly impaired PFS compared to patients without ascites (2.7 months vs 5.3 months, *p* = 0.014, Fig. 4A). The OS of patients with splenomegaly or CT-detected varices did not differ significantly from patients without splenomegaly or varices (Fig. 4B and C).

## Discussion

To the best of our knowledge, this is the first study investigating the impact of CSPH on patients with HCC undergoing immunotherapy. More than half of the included patients had CSPH according to the PH score. Furthermore, non-invasive assessment of PH identified the subgroup of patients with significantly impaired OS.

For patients with HCC undergoing tumor resection, PH has been identified as a highly predictive factor for the risk of postoperative liver decompensation [13]. Thus, the current EASL guidelines recommend to take PH into account for treatment decision making in patients with early-stage HCC [3]. However, the potential influence on the survival outcome varied in different studies on these patients [13, 18]. Moreover, CSPH has been identified as a relevant prognostic factor in patients with HCC undergoing transarterial chemoembolization (TACE) in several studies as well [14, 19, 20]. However, CSPH in patients with unresectable HCC in intermediate or advanced stages is not an established prognostic factor in the current guidelines so far.

A few studies have investigated the potential role of sorafenib treatment on existing PH. Firstly observed in preclinical animal studies, a beneficial effect on portal venous flow in magnetic resonance imaging and Doppler ultrasonography has been reported in patients with HCC and sorafenib treatment [21–24]. Furthermore, preliminary results indicate a potential decrease of HVPG in these patients during sorafenib treatment [25]. In this study, about one third of the patients showed a decrease of ≥ 20% from baseline. Those initial results have been repeated in a small external validation [26]. However, the authors argue that the effect of sorafenib in patients with PH without HCC remains to be investigated.

The rationale behind considering sorafenib as influential on portal pressure is the inhibition of VEGF-mediated angiogenesis [27]. Among the novel immunotherapy agents, particularly the combined use of atezolizumab and bevacizumab could lead to the same effect, as bevacizumab inhibits VEGF-mediated angiogenesis as well [27]. In the IMbrave150 trial, the number of gastrointestinal bleeding events due to increased portal pressure was higher in the patient group treated with atezolizumab and bevacizumab compared to patients allocated to the sorafenib group [9]. Additionally, a systematic review including phase II studies which evaluated bevacizumab monotherapy in HCC reported a gastrointestinal bleeding rate of 10% (predominantly due to esophageal varices, [10]). Thus, assessing the risk of PH-related bleeding in patients undergoing immunotherapy is of utmost importance [11].

The scarce literature on the prognostic role of PH in patients with unresectable HCC is most likely due to the fact that invasive measurement of the hepatic venous pressure gradient (HVPG) through a transjugular approach in order to assess PH directly is no standard part in the diagnostic work-up of patients with HCC. However, several studies have shown that non-invasive measurement of PH is feasible in clinical routine [16, 28–30]. In this study, we decided to use the PH score for the estimation of CSPH [16]. This score has several advantages: First, it does not require any additional data than the routinely acquired CT data. Second, it offers a clear definition of the included parameters and is therefore easy applicable in daily radiological routine. Other scores like the score system proposed by Iranmanesh et al. are in comparison hard to apply routinely as they comprise organ segmentation which is labor intensive and time consuming [28]. However, organ volume of spleen and liver for PH estimation could become more important clinically through automated organ segmentation based on artificial intelligence-based methods. Those methods have proven their feasibility in initial results and could become broadly available in the near future [31–33]. Thus, these tools could solve the ongoing discussion on splenomegaly definition as several cut-off values have been proposed for patients with HCC.

Furthermore, it remains unclear which plane for estimating splenic volume is the most suitable one: In this study, we measured the craniocaudal diameter, which has been used in the original study on the PH score and seems to be most suitable in patients with liver cirrhosis [34]. However, other studies on the role of CSPH measured the spleen size in axial plane [14, 19, 20]. Moreover, even though there is high correlation of single dimension measurements with splenomegaly [34], these measurements can only be estimates of true splenic volume and might not be representative in some cases. With the above-mentioned automated volumetry, those discussions could become obsolete.

In this study, ascites was the most relevant prognostic factor and an independent predictor of the patients’ survival outcome. This is in line with previous results on the prognostic influence of ascites prior to TACE [14]. However, similar to the problem of how to define splenomegaly, ascites is currently mostly estimated using eye-balling methods and follows no strict quantification. Therefore, purely visual estimation of volume is highly examiner-dependent and accurate manual volumetry is not feasible in clinical routine as it is even more time-consuming than manual liver and spleen segmentation. Again, artificial intelligence-assisted automated quantification could become relevant in this case in the near future.

This study has several limitations. First and foremost, this study was conducted in a retrospective manner and included only a limited number of patients. However, this dataset was well investigated and only patients with complete clinical, laboratory, and imaging data were included. Second, CSPH was assessed non-invasively using the PH score published by Kihira et al. as surrogate. True HVPG measurements were not obtained in the patients. However, due to its invasive nature, HVPG measurements via a transjugular approach are not part of the standard work-up of HCC patients and surrogate parameters deduced from cross-sectional imaging are an alternative feasible in all patients. Third, patients treated with various immunotherapeutic agents were included and no agent-specific sub-analyses were performed. Nevertheless, this shows a “real-life” clinical setting, particular as until 2021 and the positive IMbrave150 trial no standardized immunotherapy was part of the recommendations for HCC patients with advanced stage or failure of other treatment modalities [9]. Future studies should validate our results in subgroups of various immunotherapeutic agents and treatment lines.

## Conclusion

CSPH assessed non-invasively using routine CT data was identified as an independent prognostic factor in patients with HCC and immunotherapy. Hence, CSPH should be considered as highly relevant in these patients. Therefore, it might function as an additional imaging biomarker to detect high-risk patients with poor survival and possibly for treatment decision making.

## Data Availability

Data cannot be shared publicly because of institutional and national data policy restrictions since the data contain potentially identifying patient information. Data are available upon request from the Johannes Gutenberg University Mainz Medical Center (contact via https://www.radiologie-sekretariat@unimedizinmainz.de) for researchers who meet the criteria for access to confidential data (please provide the manuscript title with your enquiry).

## Abbreviations

AFP: Alpha-fetoprotein
BCLC: Barcelona Clinic Liver Cancer
Cis: Confidence intervals
CSPH: Clinically significant portal hypertension
ECOG: Eastern Cooperative Oncology Group
HCC: Hepatocellular carcinoma
HRs: Hazard ratios
INR: International Normalized Ratio
IQR: Interquartile range
OS: Overall survival
PFS: Progression-free survival
TACE: Transarterial chemoembolization
TLs: Target lesions
